# An inflammation-based cumulative prognostic score system in patients with diffuse large B cell lymphoma in rituximab era

**DOI:** 10.1186/s12885-017-3931-z

**Published:** 2018-01-02

**Authors:** Feifei Sun, Jia Zhu, Suying Lu, Zijun Zhen, Juan Wang, Junting Huang, Zonghui Ding, Musheng Zeng, Xiaofei Sun

**Affiliations:** 1 State Key Laboratory of Oncology in South China, Collaborative Innovation Center for Cancer Medicine, Sun Yat-sen University Cancer Center, NO.651 of Dongfeng East Road, Guangzhou, 510060 China; 2 Department of Pediatric Oncology, Sun Yat-sen University Cancer Center, NO.651 of Dongfeng East Road, Guangzhou, 510060 China; 3 Department of Biochemistry and Molecular Biology, Mayo Clinic Scottsdale, 13400 East Shea Boulevard, Scottsdale, AZ 85259 USA

**Keywords:** Diffuse large B-cell lymphoma, Rituximab, Inflammation, Score system, Prognostic

## Abstract

**Background:**

Systemic inflammatory parameters are associated with poor outcomes in malignant patients. Several inflammation-based cumulative prognostic score systems were established for various solid tumors. However, there is few inflammation based cumulative prognostic score system for patients with diffuse large B cell lymphoma (DLBCL).

**Methods:**

We retrospectively reviewed 564 adult DLBCL patients who had received rituximab, cyclophosphamide, doxorubicin, vincristine and prednisolone (R-CHOP) therapy between Nov 1 2006 and Dec 30 2013 and assessed the prognostic significance of six systemic inflammatory parameters evaluated in previous studies by univariate and multivariate analysis:C-reactive protein(CRP), albumin levels, the lymphocyte-monocyte ratio (LMR), the neutrophil-lymphocyte ratio(NLR), the platelet-lymphocyte ratio(PLR)and fibrinogen levels.

**Results:**

Multivariate analysis identified CRP, albumin levels and the LMR are three independent prognostic parameters for overall survival (OS). Based on these three factors, we constructed a novel inflammation-based cumulative prognostic score (ICPS) system. Four risk groups were formed: group ICPS = 0, ICPS = 1, ICPS = 2 and ICPS = 3. Advanced multivariate analysis indicated that the ICPS model is a prognostic score system independent of International Prognostic Index (IPI) for both progression-free survival (PFS) (*p* < 0.001) and OS (*p* < 0.001). The 3-year OS for patients with ICPS =0, ICPS =1, ICPS =2 and ICPS =3 were 95.6, 88.2, 76.0 and 62.2%, respectively (*p* < 0.001). The 3-year PFS for patients with ICPS = 0–1, ICPS = 2 and ICPS = 3 were 84.8, 71.6 and 54.5%, respectively (*p* < 0.001).

**Conclusions:**

The prognostic value of the ICPS model indicated that the degree of systemic inflammatory status was associated with clinical outcomes of patients with DLBCL in rituximab era. The ICPS model was shown to classify risk groups more accurately than any single inflammatory prognostic parameters. These findings may be useful for identifying candidates for further inflammation-related mechanism research or novel anti-inflammation target therapies.

## Background

Diffuse large B-cell lymphoma (DLBCL) is the most common subtype of non-Hodgkin lymphoma (NHL), representing 30–40% of all lymphomas. It is an aggressive lymphoma with heterogeneous clinicopathological, immunephenotypic, genetic features and various clinical outcomes [1–4]. Although the addition of rituximab (R) to conventional CHOP or CHOP-like regimens has significantly improved survival, approximately 30% of DLBCL patients fail chemotherapy [5]. The International Prognostic Index (IPI) based on age, performance status (PS), Ann Arbor stage, number of extranodal lesions and serum lactate dehydrogenase (LDH) level is a standard prognostic scoring system for predicting the clinical outcomes of patients with DLBCL. But in rituximab era, the ability of the IPI to predict prognosis has declined [6]. A variety of molecular biomarkers and gene signatures with prognostic significance have been identified in DLBCL patients [1–4]. However, molecular markers and gene signatures are expensive, technically challenging, and not routinely available in many undeveloped countries. Therefore, cheap and easily accessible prognostic markers which might help to increase prognostic accuracy are needed.

Malignant and inflammation are closely linked. Inflammatory processes have been identified to play an important role in the pathogenesis of lymphoma [7–9]. Pro-inflammatory cytokines (or chemokines) and inflammatory cells and in tumor microenvironment were proved to promote tumor growth, DNA damage, angiogenesis, immune suppression and to be associated with poor survival outcomes of patients [10–13]. Cytokine receptors or other factors in inflammatory pathways implicated in metastasis may be an appropriate target for malignant tumor therapy [14–19]. Circulating inflammatory parameter(including C-reactive protein (CRP), albumin levels, the lymphocyte-monocyte ratio (LMR), the neutrophil-lymphocyte ratio (NLR), the platelet-lymphocyte ratio (PLR) or fibrinogen levels) was associated with a poor prognosis in many types of malignant tumor patients including DLBCL [1, 20–27]. Several inflammation-based cumulative prognostic score systems were established for various solid tumors [28–31], but there is few inflammation-based cumulative prognostic score system for predicting survival of patients with diffuse large B cell lymphoma. Therefore, this retrospective study aimed to develop a novel inflammation-based cumulative prognostic score system for predicting survival of patients with diffuse large B cell lymphoma in rituximab era. The system may be useful for identify candidates for further inflammation related research and clinical trial of novel anti-inflammation drug.

## Methods

### Patients

We reviewed the records of 839 patients diagnosed with DLBCL according to the 2008 World Health Organization (WHO) criteria [32] at the Sun Yat-Sen University Cancer Center of China between November 1, 2006 and December 30, 2013. The data for 564 patients who received R-CHOP therapy as first-line treatment were analyzed. Clinicopathological parameters included gender, age, Ann Arbor stage, number of extranodal sites, Eastern Cooperative Oncology Group performance status (ECOG PS) and LDH level. Ann Arbor stage was made according bone marrow findings, computed tomography (CT) scans of the thorax, abdomen, and pelvic cavity, or whole body positron emission tomography/computed tomography (PET/CT) scans before treatment. The IPI was evaluated based on Ann Arbor stage, ECOG PS, serum LDH and numbers of extranodal sites. The laboratory data, including lymphocyte, neutrophil and platelet counts were obtained 1–3 days before first chemotherapy. Serum levels of CRP, albumin and fibrinogen were obtained 1–7 days before first chemotherapy. The exclusion criteria included: 1.patients treated with chemotherapy other than R-CHOP as first-line therapy; 2.patients with primary central nervous system (CNS) lymphoma; 3.patients with immunodeficiency virus infection; 4.Patients whose dose was reduced >20%; 5.Patients who did not complete their course of R-CHOP; 6.patients with clinical evidence of acute infection or chronic inflammatory disease. A total of 275 cases were excluded, including 14 cases with primary CNS DLBCL, 200 cases who received chemotherapy regimens other than R-CHOP for first-line therapy, 15 cases who did not complete their course of R-CHOP treatment, 3 cases whose therapy dose was reduced >20% and 43 cases with acute infection or chronic inflammatory disease.

### Treatment

Patient received standard R-CHOP therapy as first-line treatment [375 mg/m^2^ rituximab on day 1, 750 mg/m^2^ cyclophosphamide on day 2, 50 mg/m^2^ doxorubicin on day 2, and 1.4 mg/m^2^ (maximum 2.0 mg/body) vincristine on day 2 and 100 mg/d prednisolone on days 2–6 for 21 days per cycle] for all DLBCL patients regardless of Ann Arbor stage. Patients who had disease progression at any time or who did not achieve partial response after 4 cycles received salvage therapy. Patients in this group received R-CHOP therapy for 2 to 8 cycles (the median was 6 cycles) as first-line treatment. Residual disease, extranodal disease, or primary bulky disease was treated by radiotherapy followed by chemotherapy.

### Statistical analysis

PFS was defined from the date of diagnosis to first lymphoma progression or death from any cause, or censored at the date of last follow-up for the patients who were alive and did not have lymphoma progression. OS was defined from the date of diagnosis to death from any cause, or censored at the date of last follow-up for the patients who were alive. The optimal cut-off values for six biomarkers (CRP, albumin levels, LMR, NLR, LPR and fibrinogen levels) for predicting OS were determined using time-dependent operating characteristic (ROC) analysis, which was performed by ‘survival ROC package’ in R, version 3.3.3 (http://www.r-project.org/). The primary end point was OS, predict.time = 3 years, and the maximal Youden index (Youden index = sensitivity + specificity-1) was chosen to set optimal cut-off value. Other statistical analysis was performed with SPSS 17.0 software. The log-rank test was used to assess univariate associations between prognostic parameters and survival. The Cox proportional hazards model was used for multivariate analysis. A *P* value of less than 0.05 was regarded as statistically significant. Survival curves were constructed using the Kaplan–Meier method.

## Results

### Patients’ characteristics

A total of 564 newly diagnosed DLBCL patients who received R-CHOP regimens were analyzed, included 381(67.6%) male and 183(32.4%) female with a median age of 53 years (range 18–88 years). The Ann Arbor tumor stage distribution was as follows: stage I: 114 (20.2%) patients, stage II: 196(34.8%) patients, stage III: 129(22.9%) patients and stage IV: 125 (22.2%) patients. After a median follow-up time of 31.5 months, 139 patients relapsed or progressed in total and 96 patients died during or after first-line chemotherapy. Patients’ characteristics are summarized in Table 1.

**Table 1:** Univariate analysis of clinical parameters

	Number (%)	Univariate analysis			
		3Y–PFS (%)	*P* value	3Y–OS (%)	*P* value
Gender					
Male	312(55.3%)	74.4%	0.101	80.4%	0.017
Female	252(44.7%)	78.0%		92.0%	
Age, years					
≤ 60	381(67.6%)	78.6%	0.062	88.3%	0.002
> 60	183(32.4%)	70.2%		72.1%	
Ann Arbor stage					
I,II	310(55.0%)	85.6%	<0.001	91.8%	<0.001
III,IV	254(45.0%)	64.4%		72.8%	
LDH(U/L)					
≤ 245	337(59.8%)	84.8%	<0.001	91.6%	<0.001
> 245	227(40.2%)	62.8%		70.6%	
ECOG PS					
0,1	466(82.6%)	79.9%	<0.001	86.3%	<0.001
≥ 2	98(17.4%)	57.2%		68.5%	
Extranodal sites					
≤ 1	416(73.8%)	80.3%	<0.001	88.2%	0.017
> 1	148(26.3%)	62.5%		67.2%	
IPI					
L	309(54.8%)	94.1%	<0.001	87.6%	<0.001
LI	96(17.0%)	84.5%		72.5%	
HI	96(17.0%)	64.3%		57.0%	
H	63(11.2%)	55.4%		51.5%	
CRP (mg/L)					
≤ 8.6	357(63.3%)	83.7%	<0.001	91.2%	<0.001
> 8.6	207(36.7%)	64.0%		70.9%	
Albumin levels (g/L)					
< 41.5	266(47.2%)	67.0%	<0.001	72.8%	<0.001
≥ 41.5	298(52.8%)	83.9%		92.3%	
LMR					
≤ 2.7	348(61.7%)	63.2%	<0.001	70.0%	<0.001
> 2.7	216(38.3%)	83.9%		91.4%	
NLR					
≤ 4.6	436(77.3%)	82. 3%	<0.001	89.0%	<0.001
> 4.6	128(22.7%)	60.9%		68.8%	
PLR					
≤ 187.4	389(69.0%)	81.2%	<0.001	87.9%	<0.001
> 187.4	175(31.0%)	69.1%		76.6%	
Fibrinogen levels (g/L)					
≤ 3.8	359(63.6%)	82.9%	0.001	88.3%	0.001
> 3.8	205(36.4%)	68.8%		76.2%	

Abbreviations: *3Y–PFS* 3-year progression-free survival, *3Y–OS* 3-year overall survival, *LDH* Lactate dehydrogenase, *ECOG PS* Eastern Cooperative Oncology Group performance status, *IPI* International Prognostic Index, *L* Low, *LI* Low-intermediate, *HI* High-intermediate, *H* High, *CRP* C-reactive protein, *LMR* Lymphocyte-to-monocyte Ratio, *NLR* Neutrophil-to- lymphocyte ratio, *PLR* Platelet-to- lymphocyte ratio

### Cut-off values

First, we conducted ROC analysis to determine the optimal cut-off values of all inflammatory parameters for predicting OS as described in method above. The optimal cut-off value for CRP was 8.6 mg/L (sensitivity 66.99% and specificity 67.07%, AUC value 0.703, 95% CI 0.649–0.760, *P* < 0.001). The optimal cut-off value of the albumin levels was 41.5 g/L (sensitivity 75.78% and specificity 58.61%, AUC value 0.682, 95% CI 0.614–0.731, *P* < 0.001). The optimal cut-off value for the LMR was 2.7 (sensitivity 68.21% and specificity 67.96%, AUC value 0.704, 95% CI 0.644–0.759, *P* < 0.001). The optimal cut-off value for the NLR was 4.6 (sensitivity 44.37% and specificity 82.75%, AUC value 0.644 95% CI 0.574–0.700, *P* < 0.001). The optimal cut-off value for the PLR was 187.4(sensitivity 46.20% and specificity 72.30%, AUC value 0.587, 95% CI 0.520–0.652, *P* = 0.011). The optimal cut-off value for the fibrinogen was 3.8 g/L (sensitivity 54.20% and specificity 69.20%, AUC value 0.601, 95% Cl 0.529–0.685, *P* = 0.007).

### The novel inflammation-based cumulative prognostic score system (ICPS)

**Fig. 1:**
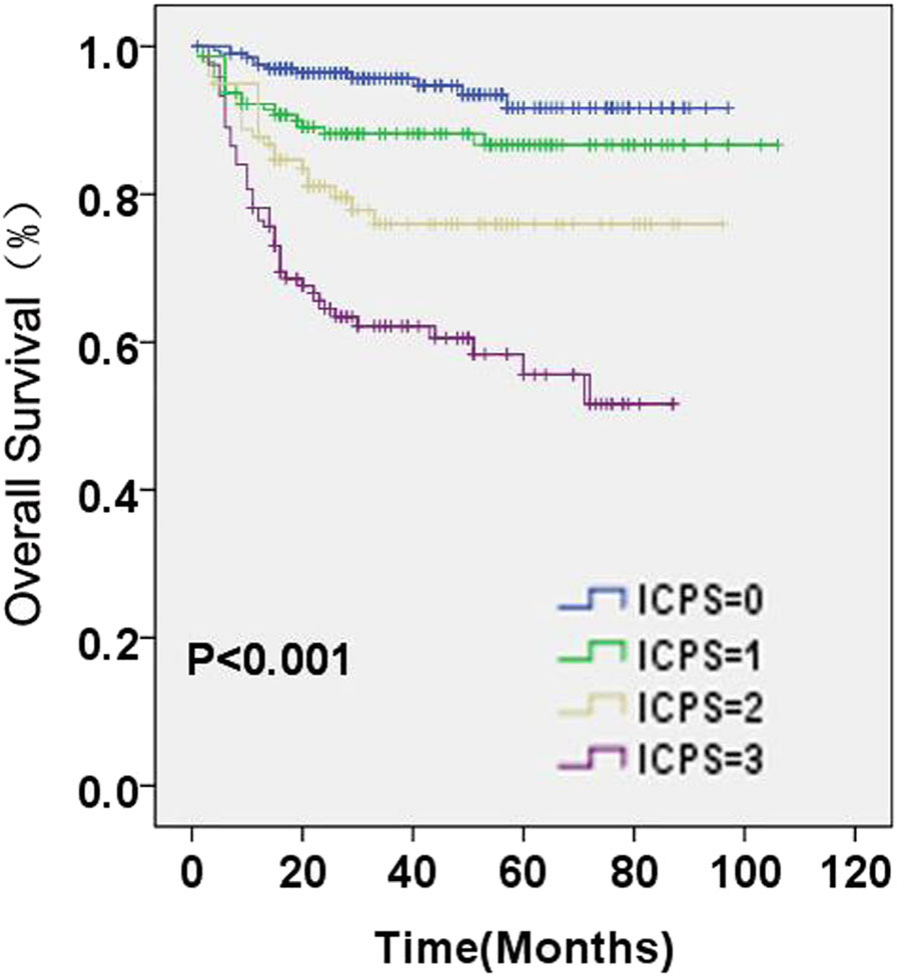
Overall survival (OS) of patients based on inflammation-based cumulative prognostic score (ICPS) system. The ICPS system classified patients into four OS risk groups (ICPS = 0, ICPS = 1, ICPS = 2 and ICPS = 3)

**Fig. 2:**
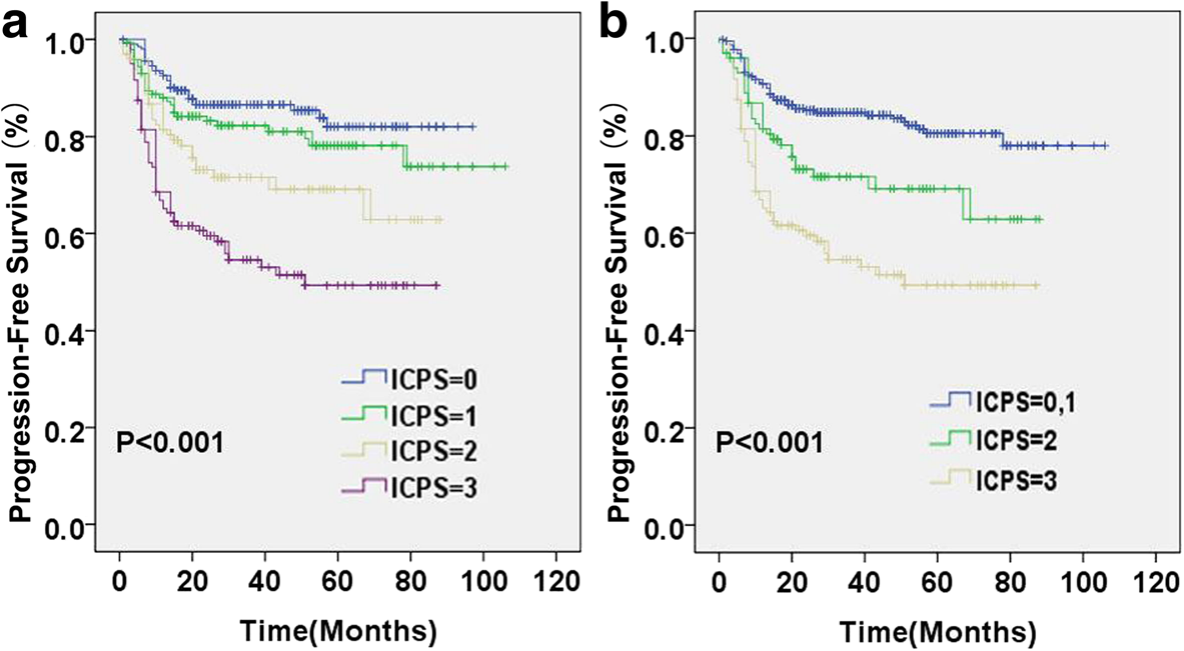
Progression-free survival (PFS) of patients based on inflammation-based cumulative prognostic score (ICPS) system. **a** The ICPS system classified patients into four PFS risk groups (ICPS = 0, ICPS = 1, ICPS = 2 and ICPS = 3); (**b**) The ICPS system classified patients into three PFS risk groups (ICPS = 0–1, ICPS = 2 and ICPS = 3)

In a univariate analysis, sex, age, localized/advanced stage, EN number, LDH, ECOG PS, IPI risk groups, CRP, albumin levels, LMR, NLR, LPR and fibrinogen levels were all significantly associated with PFS and OS (Table 1). Sex was significantly associated with OS but not PFS. Multivariate cox regression analysis for various inflammatory parameters showed that CRP, albumin levels and LMR were independent risk factors for OS, while NLR, LPR and fibrinogen levels were not (Table 2). Based on these three independent inflammatory adverse factors (CRP > 8.6 mg/L, albumin < 41.5 g/L and LMR ≤ 2.7), we constructed a novel inflammatory-based prognostic model by combing these three prognostic variables. Table 3 shows the hazard ratio (HR) and regression coefficient (β) for OS of each significant inflammatory marker. The regression coefficient (β) for OS of each significant inflammatory marker was calculated from the Cox regression eq. (HR = e^β^). Because the HR for OS of each significant inflammatory marker is very close, we put the same weight on each factor in the prognostic-score model. The sum of the points allotted correlates with the following risk groups: group ICPS = 0 (*n* = 202, 35.8%), no inflammatory adverse factors; group ICPS = 1 (*n* = 144, 25.5%), 1 factor; group ICPS = 2 (*n* = 99, 17.6%), 2 factors; and ICPS = 3 (*n* = 119, 21.1%), 3 factors. In an advanced multivariate analysis adjusted for IPI risk factors (age, localized/advanced stage, EN number, LDH and ECOG PS), ICPS is an independent prognostic factor for both PFS and OS (Table 4). And ICPS is also a prognostic score system independent of IPI score (Table 5). The proportion of patients in each ICPS group and the associated hazard ratios in survival analysis are presented in Table 6. The relative risk of group ICPS =0, ICPS =1, ICPS =2 and ICPS =3 for OS was 1.000, 2.270(1.063–4.847), 4.395(2.118–9.118) and 8.645(4.482–16.676), respectively. The relative risk of group ICPS =0, ICPS =1, ICPS =2 and ICPS =3 for PFS was 1.000, 1.409(0.838–2.369), 2.241(1.333–3.767) and 3.957(2.518–6.216), respectively. Figures 1 and 2 shows Kaplan-Meier curves for OS and PFS stratified according to the ICPS group, respectively. The 3-year OS rates for patients with ICPS =0, ICPS =1, ICPS =2 and ICPS =3 were 95.6, 88.2, 76.0 and 62.2%, respectively(Fig. 1). It showed that patients with lower ICPS had significantly better OS (*p* < 0.001). The ICPS model can significantly distinguish between any neighboring two risk groups. The 3-year PFS rates for patients with ICPS = 0, ICPS = 1, ICPS = 2 and ICPS = 3 were 86.5, 82.3, 71.6 and 54.5%, respectively (Table 6, Fig. 2a). The result showed that the PFS difference between group ICPS = 0 and ICPS = 1 is insignificance, but the difference between any two group of ICPS = 0, ICPS = 2 and ICPS = 3 is significant. So we merged patients with ICPS = 0 and ICPS = 1 to one group (ICPS = 0–1). In PFS analysis, we could classify patients to three risk groups (group ICPS = 0–1, ICPS = 2 and ICPS = 3) (Fig. 2b). The 3-year PFS rates for patients with ICPS = 0–1, ICPS = 2 and ICPS = 3 were 84.8, 71.6 and 54.5%, respectively. The PFS difference between any two of three risk groups is significant (*p* < 0.001).

**Table 2:** Multivariate analysis of various inflammatory parameters

Inflammatory parameters	OS		PFS	
	HR (95% CI)	*P*-value	HR (95% Cl)	*P*-value
Albumin levels(<41.5 g/L)	2.911(1.533–5.525)	0.001	2.025(1.278–3.208)	0.003
LMR(≤2.7)	1.966(1.100–3.513)	0.022	1.688(1.062–2.684)	0.027
CRP(>8.6 mg/L)	1.804(0.983–3.310)	0.037	1.588(0.993–2.541)	0.054
NLR(>4.6)		0.306		0.183
PLR(>187.4)		0.604		0.205
Fibrinogen levels (>3.8 g/L)		0.800		0.266

Abbreviations: *OS* Overall survival, *PFS* Progression-free survival, *HR* Hazard Ratio, 95% Cl, 95% confidence limits, *LMR* Lymphocyte-to-monocyte Ratio, *CRP* C-reactive protein, *NLR* Neutrophil-to- lymphocyte ratio, *PLR* Platelet-to- lymphocyte ratio

**Table 3:** The hazard ratio (HR) and regression coefficient (β) for OS of each significant inflammatory marker

Factor	HR	β(HR = e^β^)	score
Albumin levels < 41.5 g/L	2.245(1.366–3.691)	0.81	1
LMR ≤ 2.7	2.019(1.225–3.328)	0.70	1
CRP > 8.6 mg/L	1.842(1.102–3.078)	0.61	1

Abbreviations: *OS* Overall survival, *HR* Hazard Ratio, *LMR* Lymphocyte-to-monocyte Ratio, *CRP* C-reactive protein

**Table 4:** Multivariate analysis of the inflammation-based cumulative prognostic score (ICPS) and IPI risk factors

Characteristic	OS		PFS	
	*P*-value	HR (95% Cl)	*P*-value	HR (95% Cl)
Age(>60y)	<0.001	2.493 (1.657–3.749)	0.250	1.228(0.865–1.745)
Ann Arbor stage(III,IV)	0.533	1.185(0.695–2.022)	0.295	1.254(0.821–1.914)
LDH(>245 U/L)	0.042	1.690(1.020–2.800)	0.018	1.642(1.087–2.479)
ECOG PS(≥2)	0.411	1.230(0.751–2.012)	0.022	1.579(1.069–2.330)
Extranodal sites(>1)	0.013	1.803(1.134–2.868)	0.187	1.305(0.879–1.937)
ICPS ≥ 2	0.001	2.476 (1.483–4.136)	0.019	1.611(1.080–2.404)

Abbreviations: *OS* Overall survival, *PFS* Progression-free survival, *HR* Hazard Ratio, 95% Cl, 95% confidence limits, *LDH* Lactate dehydrogenase, *ECOG PS* Eastern Cooperative Oncology Group performance status, *IPI* International Prognostic Index, *Hi* High-intermediate, *H* High, *ICPS* Inflammation-based cumulative prognostic score

**Table 5:** Multivariate analysis of the inflammation-based cumulative prognostic score (ICPS) and IPI score

Characteristic	OS		PFS	
	*P*-value	HR (95% Cl)	*P*-value	HR (95% Cl)
IPI (Hi, H)	<0.001	2.697 (1.652–4.405)	0.001	1.892(1.293–1.767)
ICPS ≥ 2	<0.001	3.044 (1.929–4.804)	<0.001	2.293(1.577–3.335)

Abbreviations: *OS* Overall survival, *PFS* Progression-free survival, *HR* Hazard Ratio, 95% Cl, 95% confidence limits, *IPI* International Prognostic Index, *Hi* High-intermediate, *H* High, *ICPS* Inflammation-based cumulative prognostic score

**Table 6:** Survival rate and relative risk according to risk group as defined by the ICPS system

Risk group	No.of patients (%)	3-year OS (%)	RR (95%Cl)	*P*-value	3-year PFS (%)	RR (95%Cl)	*P*-value
ICPS = 0	202(35.8)	95.6	1.000(N/A)	<0.001	86.5	1.000(N/A)	<0.001
ICPS = 1	144(25.5)	88.2	2.270(1.063–4.847)	0.034	82.3	1.409(0.838–2.369)	0.196
ICPS = 2	99(17.6)	76.0	4.395(2.118–9.118)	<0.001	71.6	2.241(1.333–3.767)	0.002
ICPS = 3	119(21.1)	62.2	8.645(4.482–16.676)	<0.001	54.5	3.957(2.518–6.216)	<0.001

Abbreviations: *3Y–OS* 3-year overall survival, *3Y–PFS* 3-year progression-free survival, *RR* Relative risk, 95% Cl, 95% confidence limits, *ICPS* Inflammation-based cumulative prognostic score

## Discussion

It is well established that systemic inflammation status is associated with poor outcome in various solid tumors and lymphomas [5, 10, 33–36]. Prognostic parameters which reflect systemic inflammatory response include CRP, albumin levels, LMR, NLR, PLR and fibrinogen levels [1, 20–27], etc. Furthermore, recent studies had indicated that inflammation-based cumulative prognostic score systems, like the Glasgow prognostic score (GPS) or advance lung cancer inflammation index (ALI), are useful prognostic models for several solid tumors [28–31]. A retrospective study aslo showed that the modified Glasgow prognostic scores (mGPS) can be used as a predictor in diffuse large B cell lymphoma treated with R-CHOP regimen [37]: patients with lower mGPS had higher complete response rate and better OS, however, the difference of PFS was statistically insignificant. So far, there is few inflammation-based cumulative prognostic score systems for predicting survival of patients with diffuse large B cell lymphoma. To overcome the lacking data, we accessed prognostic values of inflammatory prognostic parameters commonly used in publications including CRP, albumin levels, LMR, NLR, PLR and fibrinogen levels. The results of univariate and multivariate analysis showed that albumin levels, CRP and LMR are three independent prognostic factors for OS. Based on these three independent inflammatory adverse factors (CRP > 8.6 mg/L, albumin levels < 41.5 g/L and LMR ≤ 2.7), we developed a novel systemic inflammatory cumulative prognostic score system, which we called the ICPS. An advanced multivariate analysis showed that the ICPS was a prognostic system independent of IPI risk factors including age, Ann Arbor stage, number of extranodal sites, ECOG PS and serum LDH level. Compared with any single inflammatory prognostic parameters, the ICPS model was shown to classify risk groups more accurately. The ICPS model can classify patients to four risk groups for OS and three risk groups for PFS. The survival difference between any neighboring two risk groups is significant in both PFS and OS analysis. As our best knowledge, it is the best inflammatory-based prognostic score system reported in DLBCL patients so far. Patients with higher ICPS had poorer clinical outcomes, which indicated that the degree of systemic inflammatory status had indeed been associated with clinical outcomes of patients with DLBCL in rituximab era. Inflammation plays an important role in malignant tumor development. Inflammatory cytokines (including tumor necrosis factor (TNF), Interleukins (IL) and chemokines, etc.) and immune cells (including tumor-associated macrophages and tumor infiltrating lymphocytes, etc.) in tumor microenvironment contribute to tumor growth, metastasis and immunosuppression [10–13]. Functional polymorphisms of inflammatory cytokine genes have been demonstrated to be associated with lymphoma risk and outcomes [38–40]. Chemokines or other inflammatory factors implicated in metastasis are considered to be potential targets for malignant tumor therapy [41–43]. Elevated levels of serum inflammatory markers reflect the inflammatory response of the body to malignant tumors. For example, C-reaction protein(CRP)is an acute phase protein produced by the liver after cytokines stimuli. Inflammation-based cumulative prognostic score systems were shown to be associated with levels of serum chemokines in patients with lymphoma [44]. The molecular mechanism of high degree of systemic inflammation status in malignant patients is unknown, whether these patients have abnormity of inflammatory pathways awaits future investigation. If be confirmed, these inflammation-based score systems may be useful in identifying patients for further inflammation-related mechanism research or novel anti-inflammation drug or gene target therapies.

At our lab, the normal range of CRP is 0-5 mg/L, the normal range of albumin level is >35 mg/L, the normal range of lymph:mono ratio is not defined. The cut-off value of albumin level in this study is 41.5 mg/L based on ROC analysis, which falls into the normal range. Univariate analysis showed that albumin levels were positively correlated with OS and PFS, regardless of whether the cut-off value was 41.5 mg/L or 35 mg/L. However, multivariate analysis with albumin level and IPI risk factors found that (data was not shown): when the cut-off value was set at 41.5 mg/L, low albumin level would be an independent risk factor for poor outcomes (*P* = 0.002 for OS, *P* = 0.004 for PFS). When the cut-off value was set at 35 mg/L, the prognostic value of low albumin level disappeared (*P* = 0.442 for OS, *P* = 0.338 for PFS). Although albumin level 35~41.5 mg/L was in the normal range, survival of those patients were no difference with whose albumin level < 35 mg/L (3-year survival 74.7% VS.67.5%, *P* = 0.220 for OS; 69.4% VS.59.7%, *P* = 0.091 for PFS), but significantly worse than whose ≥ 41.5 mg/L(3-year survival 74.7% VS.92.1%,*P* < 0.001 for OS; 69.4% VS.83.8%,*P* < 0.001 for PFS)**.** So we defined 41.5 mg/L as cut-off value other than 35 mg/L. Most other studies of DLBCL patients used 35 mg/L as a cut-off value, which may lead to negative results. We speculate that slight decrease of serum albumin level may be caused by increase of inflammatory cytokines secretion and vascular permeability which is induced by systemic inflammatory states in lymphoma patients. While severe hypoalbuminemia may be caused mostly by malnutrition, tumor consumption, pleural or peritoneal effusion and edema, so it is independently associated with IPI risk factors. This hypothesis requires more evidence to support.

There are several limitations in this study. First, some cases chose treatment regimens without Rituximab or did not complete their course of R-CHOP treatment because the cost of Rituximab was beyond which they can afford. In order to ensure the homogeneity of treatment, these patients were excluded, which might result in a selection bias. Second, the ICPS model is based on a single-center retrospective study in South China, which has not been validated in other populations. Multicenter prospective studies are needed to determine whether it is suitable for large sample groups.

## Conclusions

In summary, we have developed a novel systemic inflammatory cumulative prognostic score system independent of the IPI in DLBCL patients treated with R-CHOP therapy. The score system based on serum CRP, albumin levels and peripheral LMR, which we called the ICPS. The ICPS model can classify patients into four risk groups with significantly different survival outcomes. The research indicated that the degree of systemic inflammatory status was associated with clinical outcomes of patients with DLBCL receiving R-CHOP therapy. Additional prospective multicenter studies are needed to confirm the clinical potential of the ICPS as prognostic system in patients with DLBCL. The molecular abnormity of high degree of systemic inflammation status in these DLBCL patients awaits future investigation. The ICPS model may be useful in identifying patients for further inflammation-related mechanism research or novel anti-inflammation gene or drug target therapies.

## Abbreviations

CRP: C-reactive protein
DLBCL: Diffuse large B cell lymphoma
GPS: The Glasgow prognostic score
ICPS: Inflammation-based cumulative prognostic score system
IL: Interleukins
IPI: International Prognostic Index
LDH: Lactate dehydrogenase
LMR: The lymphocyte-monocyte ratio
NHLs: Non-Hodgkin’s lymphomas
NLR: The neutrophil-lymphocyte ratio
OS: Overall survival
PFS: Progression-free survival
PLR: The platelet-lymphocyte ratio
PS: Performance status
ROC: Operating characteristic
TNF: Tumor necrosis factor
